# Novel variant of reversed midgut rotation – retro-arterial proximal jejunum and transverse colon: a case report and review of the literature

**DOI:** 10.1186/s13256-018-1802-0

**Published:** 2018-09-14

**Authors:** Dominik Deniffel, Sebastian M. Goerke, Ernst J. Rummeny, Jörg Laubenberger

**Affiliations:** 1 Department of Diagnostic and Interventional Radiology, Klinikum rechts der Isar, Technical University of Munich, Ismaninger Str. 22, 81675 München, Germany; 2 Department of Radiology, Ortenau Klinikum Offenburg-Gengenbach, Ebertplatz 12, 77654 Offenburg, Germany

**Keywords:** Intestinal malrotation, Reverse rotation variant, Midgut development, Embryology, Anatomical anomaly, Rare congenital disorder, Small bowel obstruction

## Abstract

**Background:**

Reversed rotation of the midgut is the rarest variation of midgut malrotations, which are congenital disorders that result from aberrant rotation and fixation of the midgut during embryological development. Common complications of these disorders are small bowel obstruction by volvulus or peritoneal bands, usually occurring in early infancy.

**Case presentation:**

A 23-year-old Caucasian woman presented with recurrent abdominal pain. A contrast-enhanced multidetector computed tomography study revealed a novel variant of reversed rotation of the midgut. Besides the specific finding of a retro-arterial transverse colon, we also found the proximal jejunum to cross posterior to the mesenteric root, a variation that has not been reported in the literature so far. In this case, substantial symptomatic relief was achieved with conservative management.

**Conclusions:**

The hypothesis of a double reversed rotation of the pre-arterial segment of the umbilical loop around the superior mesenteric artery axis provides a possible explanation for this anomaly. There is no evidence-based consensus on the management of patients presenting with non-symptomatic or mildly symptomatic intestinal malrotations. In this case, radiologic and clinical presentations excluded acute small bowel obstruction, and surgical intervention was avoided.

## Background

Intestinal malrotation is a rare congenital disorder, defined by an abnormal position of the bowel within the peritoneal cavity, which results from a failure in the normal course of midgut rotation and fixation during embryologic development. Depending on which stage of the physiological sequence of midgut development is stopped or disrupted, a variety of anatomic anomalies can occur, comprising non-rotation, incomplete rotation, mixed-rotation, and reversed rotation [1]. Since these anatomic variants can go unnoticed or undiagnosed without ever causing any clinical symptoms, their exact incidence is unknown. Intestinal malrotations with clinically relevant symptoms occur approximately in 1/6000 live births [2]. Reversed rotation, by far the rarest presentation of intestinal rotation and fixation anomalies, accounts for 2–4% of all malrotation cases [3].

The most common clinical manifestation of intestinal malrotation in neonates is an acute duodenal obstruction or midgut volvulus, a life-threatening condition in which the bowel and its accompanying mesenteric vessels twist around the short mesenteric pedicle resulting in bowel obstruction and ischemia. In adolescents and adult patients the incidence of volvulus declines [4] and clinical presentation is more variable [4–6], which complicates diagnosis and delays proper treatment, resulting in increased morbidity [5, 7].

In this article, we present, to the best of our knowledge, the first published case of a novel variant of reversed rotation of the midgut as a cause for recurrent abdominal pain in a young adult.

## Case presentation

A 23-year-old Caucasian woman presented to our emergency department with abdominal pain and recurrent nausea of 6 days’ duration, which had progressively worsened over the past few hours, but without vomiting. The pain, she described, was rather diffuse but more intense in the epigastric region. She denied reflux, diarrhea, urinary symptoms, and fever. She reported that she had been having intermittent problems with diffuse abdominal pain throughout her adult life, but usually milder than this current episode.

Regarding her medical history, she had suffered from a jejunal atresia in her left-upper abdomen which required surgical treatment in her neonatal period. It was initially treated with a Bishop–Koop side-to-side jejunojejunostomy with chimney. The stoma was reversed approximately 7 months later.

A physical examination revealed a flat and soft abdomen with a big scar across her upper abdomen, without any evidence of a hernia. Abdominal palpation revealed a mild tenderness in her lower abdomen, particularly in the right lower quadrant. No abdominal masses were palpable. Bowel sounds were normal. A laboratory examination (complete blood count, electrolytes, C-reactive protein, liver, pancreatic, thyroid, and renal function) revealed no abnormality.

**Fig. 1:**
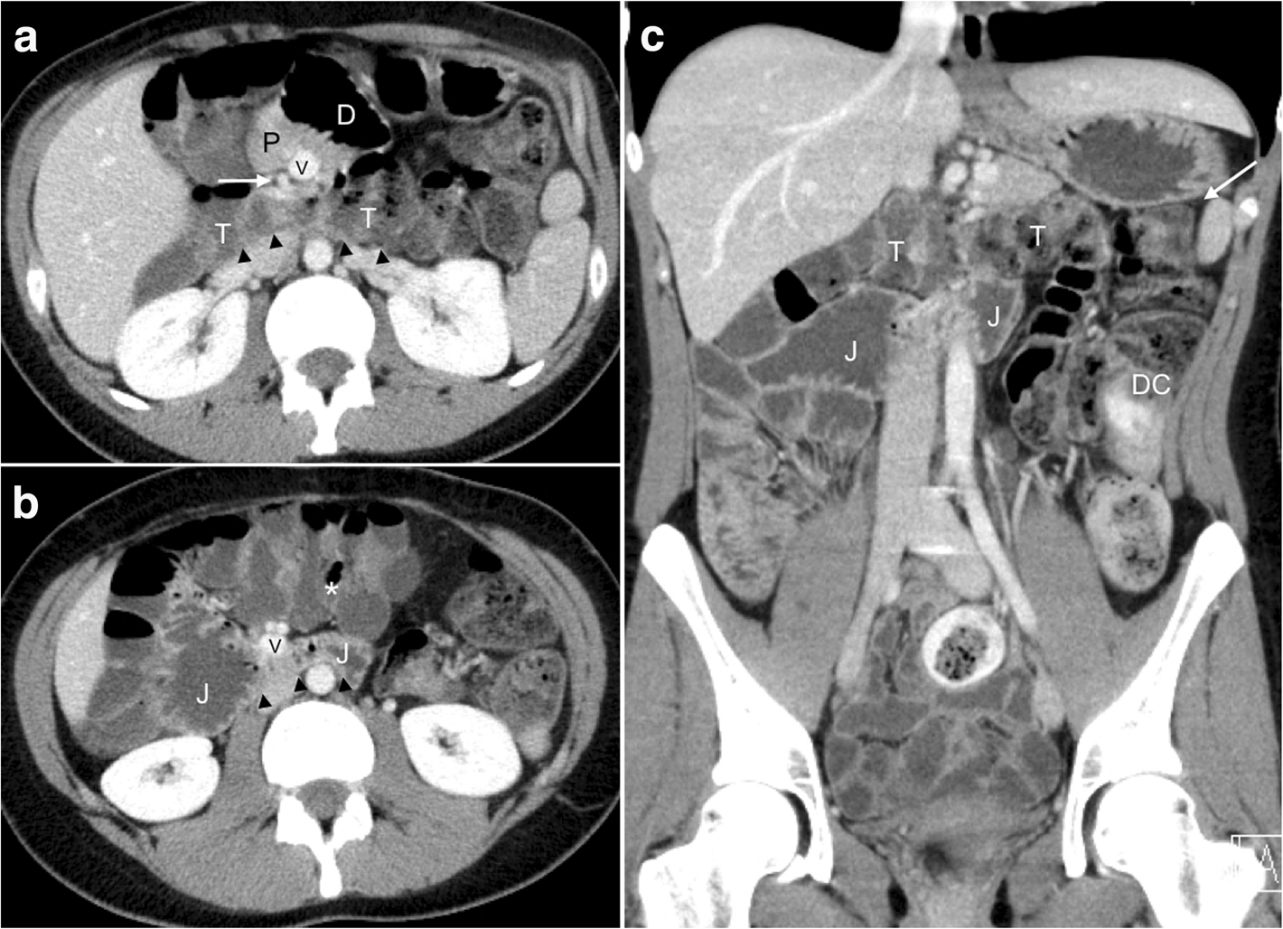
Contrast-enhanced multidetector computed tomography study. **a** At the level of the pancreatic head (*P*): retro-arterial course (*arrowheads*) of the transverse colon (*T*) dorsal to the mesenteric vessels. Note the inverted relationship of the superior mesenteric vein (*V*) and superior mesenteric artery (*white arrow*) and the aplasia of the pancreatic uncinate process. The duodenum (*D*) crosses from right to left anterior to the pancreas and the mesenteric vessels. **b** At the level of the posterior-inferior margin of the right hemiliver: retro-arterial course (*arrowheads*) of the proximal jejunum (*J*) from left to right, sandwiched between the superior mesenteric vessels and the aorta. Note the aberrant position of the cecum and the ileocecal valve (*) close to the midline. **c** Coronal reformatted image: normal position of the left colic flexure (*white arrow*) and the descending colon (*DC*). The relationship between the transverse colon (*T*) and the jejunum (*J*), both in a retro-arterial position, is demonstrated. Several small bowel loops are noted in the lower abdomen

**Fig. 2:**
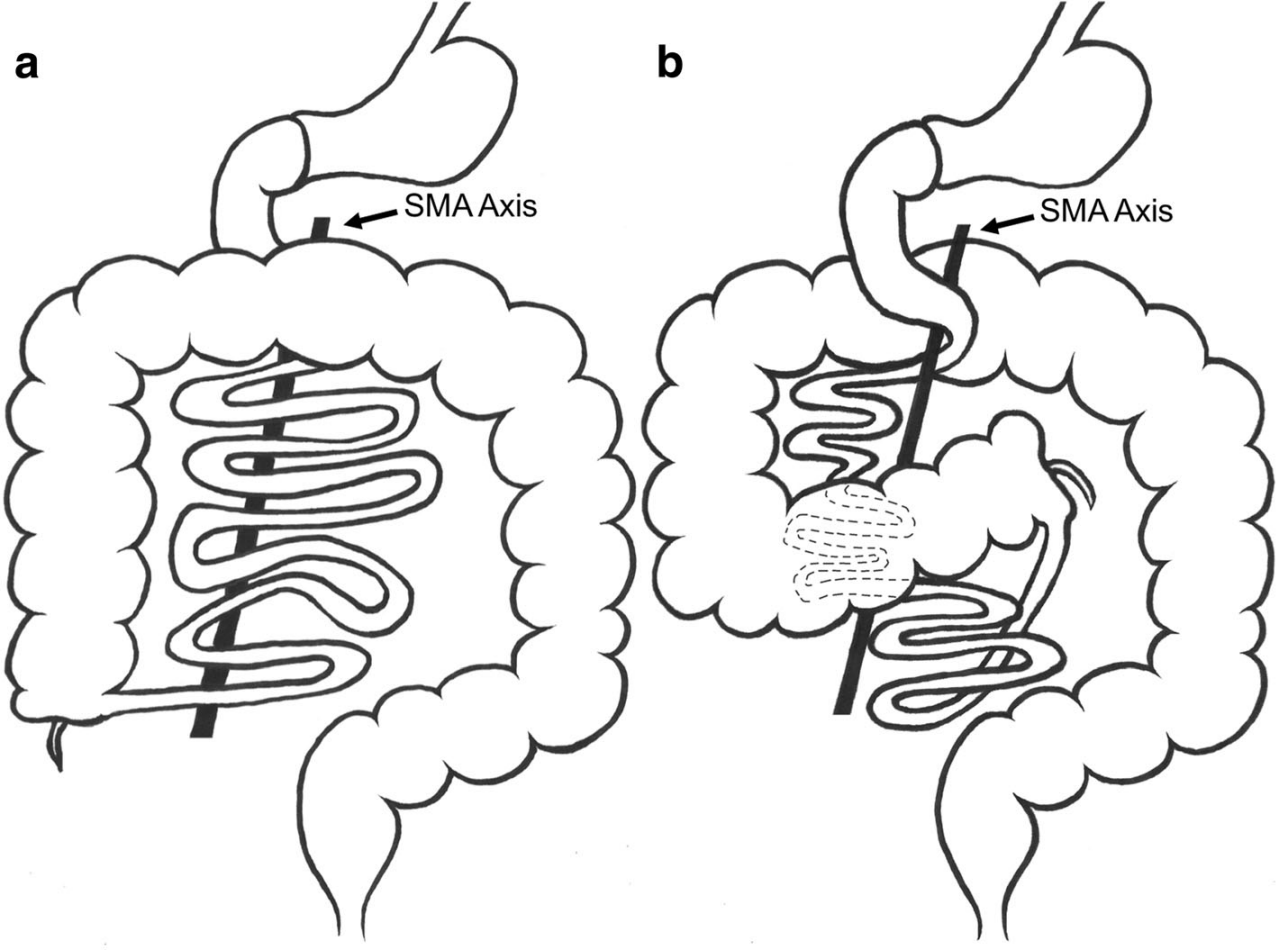
Anatomic illustrations of the gastrointestinal tract. **a** After normal midgut rotation: the duodenum is mostly in a retroarterial, and the jejunum and transverse colon are in an intraperitoneal position. **b** Anatomical situation in our present case: the transverse colon and the proximal jejunum are positioned posterior to the superior mesenteric artery axis. The ileocecal valve is found close to the midline in the mid-abdomen, whereas the hindgut is normally positioned. *SMA* superior mesenteric artery

A contrast-enhanced computed tomography (CT) scan of her abdomen with rectal contrast enema was performed. The CT scan demonstrated a markedly altered anatomy of the midgut with a rather usual course of the hindgut (Fig. 1a–c). The normal anatomy of the gastrointestinal tract and the anatomical situation in the present case are graphically illustrated in Fig. 2a and b, respectively. The duodenum crossed from right to left ventral to the superior mesenteric artery (SMA), suggesting an intraperitoneal position in all of its portions, with the duodenojejunal flexure situated slightly to the left of the midline. The proximal jejunum then crossed back to the right abdomen, posterior to the SMA, suggesting a retroperitoneal position (Fig. 1b). The following slightly dilated jejunal loops were found lying in the right abdomen. Further distally the distal jejunum/proximal ileum crossed back to the left lower abdomen, remaining in an intraperitoneal position ventral to the mesenteric root. Most of the ileal loops were situated in the left lower abdomen (Fig. 1c); the cecum was found slightly left of the midline in the umbilical region in close proximity to the ligament of Treitz (Fig. 1b), thus suggesting a narrow mesenteric pedicle. The ascending colon coursed from the right mid-abdomen toward the ileocecal pole in the left mid-abdomen. Both ascending colon and cecum remained ventral to the mesenteric root in an intraperitoneal position. The transverse colon dorsally crossed the pedicle of the SMA and the superior mesenteric vein (SMV) in a retro-arterial position (Fig. 1a), defining this anatomic midgut variation as reversed rotation, and extended to the left abdomen to continue as a normal left colic flexure and descending colon. We further noted an inverted relationship of the SMV to the SMA with the vein lying to the left of the artery and an aplasia of the uncinate process (Fig. 1a). No thickened bowel walls or peritoneal fluid were present.

CT demonstrated no evidence of frank volvulus, bowel ischemia, or acute bowel obstruction. The patient was diagnosed as having a variant of reversed intestinal rotation.

She was immediately started on intravenously administered fluids and analgesics (metamizole), which offered instant pain relief. The clinical findings did not indicate a need for immediate hospital admission or surgical intervention.

## Discussion

In the normal sequence of intestinal development, the midgut, which extends from the entrance of the bile duct into the duodenum to the last third of the transverse colon, rotates 270 degrees counterclockwise around the axis of the SMA. This rotation occurs during the fourth through to the 12th week of gestation [8].

Due to the rapid growth of the midgut, it initially extends into the extra-abdominal cavity and forms the umbilical loop, which is positioned sagittally. This process is known as physiological herniation of the midgut. Further growth of this umbilical loop is associated with a rotation of 90 degrees around the axis of the SMA in a counterclockwise direction, resulting in a horizontal position of the umbilical loop. The small intestine comes to lie to the right and the cecum to the left of the SMA. Between 8.5 and 9.0 weeks of development, the physiological umbilical hernia resolves as the abdominal cavity becomes sufficiently large. The loops of the small intestine return first from the umbilical stalk into the abdomen, while the cecum returns last, undergoing an additional 180 degrees counterclockwise rotation [8]. As a result of this, the duodenum acquires a position posterior to the SMA, the duodenojejunal junction a left-sided and the cecum a right-sided position (Fig. 2a). The broad mesenteric base runs obliquely from the inferior part of the duodenum to the ileocecal valve [9], preventing the small bowel from twisting around the SMA.

Reversed intestinal rotation was first reported in 1883 by Tscherning [10]. In 1923, Dott [11] suggested that reversed rotation occurs when the initial 90 degrees counterclockwise rotation of the umbilical loop is followed by an 180 degrees clockwise rotation, resulting in a net 90 degrees clockwise rotation. Estrada [12] further classified reversed intestinal rotation into two subtypes: retro-arterial and pre-arterial. In the more common retro-arterial subtype the migration into the peritoneal cavity begins with the cecum, passing to the right and posterior to the SMA. As a consequence, the transverse colon lies behind the duodenum and is separated from it by the SMA. The duodenum remains intraperitoneal, anterior to the SMA. In the less common pre-arterial subtype, the pre-arterial segment is thought to return first into the peritoneal cavity, lying anterior to the SMA in the left abdomen. The post-arterial segment then ends up in the right abdomen [12, 13].

Our case differs from previously reported reversed rotations, due to the retro-arterial course of the proximal jejunum. This unusual anatomy may be explained by a variant of the retro-arterial subtype of reversed rotation. We suggest that the post-arterial segment returned first to the abdominal cavity undergoing a clockwise rotation, characteristic of reversed rotation. The following pre-arterial segment takes an additional turn of net 360 degrees in clockwise (reverse) direction, instead of the usual 90 degrees in reversed rotations, thereby resulting in a 360 degrees loop of the pre-arterial segment around the SMA axis (Fig. 2b). This variant differs from an earlier described case with the suggested name of double reversed intestinal rotation, where only the retro-arterial segment seems to complete a 360 degree rotation around the mesenteric root [14]. In view of this latter and our variant of reversed rotation, presumably, a multitude of combinations from partial to complete reversed rotations of the pre-arterial or retro-arterial segment are imaginable, in addition to the currently believed classic appearance of reversed intestinal rotation.

Due to its extreme rarity, little is known about the pathophysiology of this condition and the exact incidence of complications is still difficult to determine. Reversed intestinal rotation has repeatedly been reported to be associated with impaired fixation and high mobility of the ascending colon and cecum, which is, therefore, prone to ileocecal volvulus [15–19]. With the cecum located in the mid-abdomen, close to the midline, a mobile cecum with a narrow mesenteric attachment was presumably also present in our patient. Other complications observed in reversed rotation anomalies are obstruction of the transverse colon by the SMA in the retro-arterial tunnel [17–21], volvulus (especially ileocecal) [15–19], and duodenojejunal obstruction. The latter may occur due to paraduodenal herniation or fibrous peritoneal bands [15, 16, 19, 20, 22, 23]. These complications require rapid diagnosis and efficient surgical management.

When intestinal malrotation is suspected, the most reliable radiologic sign is an intraperitoneal position of the D3-segment of the duodenum or, in other words, a retroperitoneal retromesenteric position of the D3-segment almost certainly excludes rotational anomalies of the midgut [24, 25]. Another sign, typically associated with intestinal malrotations, is inverted mesenteric vessels, as also seen in our case.

The therapeutic gold standard in the management of acute complications resulting from intestinal malrotations is a surgical approach [9]. The standard Ladd’s procedure, named after the American pediatrician William Edwards Ladd, consists of surgical division of obstructing fibrous peritoneal bands, the section of possible adhesions near the superior mesenteric vessels, and an appendectomy, due to the abnormal position of the cecum. The aim of the Ladd’s procedure is to relieve already present bowel obstruction and to widen the base of the mesentery to prevent midgut volvulus [9]. The surgical approaches described to correct reversed rotations further include resection and displacement of the transverse colon anterior to the mesenteric root [14] or right hemicolectomy with ileotransverse anastomosis [20]. In other cases, extensive resections or antemesenteric transposition of the transverse colon was avoided [21, 26].

As a matter of course, the choice of a surgical management is imperative when patients present with acute life-threatening complications. However, the dramatic rise of cross-sectional imaging and the incidental diagnosis of intestinal malrotations in asymptomatic or mildly symptomatic patients raise the question of adequate treatment in this patient group. The majority of pediatric surgeons recommend a surgical correction in any given case if intestinal malrotation is diagnosed [27–30]. However, a recent meta-analysis compiling data from 1980 to 2013 regarding asymptomatic intestinal malrotations drew the conclusion that, lacking multicenter and prospective data for this complex group of patients, there is no convincing evidence for or against a particular approach, so that watchful waiting should be considered an option in individual cases [31].

## Conclusions

We present a novel variant of reversed midgut rotation, which may be explained by a double reversed rotation of the pre-arterial segment of the umbilical loop around the SMA axis. In this case, the radiologic and clinical presentations excluded acute small bowel obstruction, and extensive surgical intervention was avoided.

## Abbreviations

CT: Computed tomography
SMA: Superior mesenteric artery
SMV: Superior mesenteric vein
